# Irritable Bowel Syndrome in medical students at a Peruvian university: a cross-sectional study

**DOI:** 10.3389/fmed.2024.1341809

**Published:** 2024-04-05

**Authors:** Pedro P. Quiroga-Castañeda, Iván Berrios-Villegas, Danai Valladares-Garrido, Víctor J. Vera-Ponce, J. Pierre Zila-Velasque, César Johan Pereira-Victorio, Mario J. Valladares-Garrido

**Affiliations:** ^1^Facultad de Medicina, Universidad de San Martín de Porres, Chiclayo, Peru; ^2^Escuela de Medicina, Universidad Cesar Vallejo, Trujillo, Peru; ^3^Oficina de Salud Ocupacional, Hospital Santa Rosa, Piura, Peru; ^4^Instituto de Investigación de Enfermedades Tropicales, Universidad Nacional Toribio Rodríguez de Mendoza de Amazonas, Chachapoyas, Peru; ^5^Universidad Tecnológica del Perú, Lima, Peru; ^6^Facultad de Medicina Humana, Universidad Nacional Daniel Alcides Carrión, Pasco, Peru; ^7^Red Latinoamericana de Medicina en La Altitud e Investigación (REDLAMAI), Pasco, Peru; ^8^School of Medicine, Universidad Continental, Lima, Peru; ^9^Escuela de Medicina, Universidad César Vallejo, Piura, Peru

**Keywords:** Irritable Bowel Syndrome, medical students, social factors, Peru, mental health

## Abstract

**Background:**

Irritable Bowel Syndrome has emerged as a significant public health challenge, particularly relevant in medical students due to the high demands of their studies, academic stress, and susceptibility to eating disorders. Nevertheless, conclusive evidence regarding the factors associated with Irritable Bowel Syndrome in the Latin American student population remains limited. The objective of this study was to determine the prevalence and factors associated with Irritable Bowel Syndrome in Human Medicine students at a university in northern Peru.

**Methods:**

A cross-sectional analytical study conducted in Lambayeque, northern Peru. With 403 Human Medicine students (66.5% female, 33.5% male). A simple random probabilistic sampling type was used, based on a list of students enrolled. A multivariate analysis was conducted to determine the factors associated using simple and multiple regression models. Generalized Linear Models were applied, using the Poisson distribution family, robust variance, and the academic year as a cluster.

**Results:**

The prevalence of Irritable Bowel Syndrome was 16.9% (95% CI: 13.37–20.86). The median age was 21 years, with 66.5% being female. In the multiple regression analysis, Irritable Bowel Syndrome was associated with a higher prevalence of depression (PR: 3.63; 95% CI: 1.26–10.49) and eating disorders (PR: 1.57; 95% CI: 1.01–2.43). For each additional year of age, the prevalence of Irritable Bowel Syndrome decreased by 9% (PR: 0.91; 95% CI: 0.83–0.99).

**Conclusion:**

This study reveals that approximately two out of every 10 students exhibit symptoms related to IBS, underscoring its significance in the Human Medicine student population. Furthermore, depression and eating disorders were identified as significant factors associated with IBS in students. Consequently, it is essential to focus efforts on early identification and the implementation of preventive measures to mitigate the development of this pathology, given its substantial prevalence in this context of Human Medicine students.

## Introduction

Irritable Bowel Syndrome (IBS) is a benign functional digestive disorder with significant social and economic repercussions on the daily lives of those affected by it (1). According to the Rome IV criteria, IBS is defined as the presence of recurrent abdominal pain for more than 3 days per month, during the previous 3 months, associated with two or more of the following criteria (relation to defecation, changes in stool frequency, and/or changes in stool consistency) (2). The symptoms characterizing IBS include chronic abdominal pain accompanied by abdominal distension and flatulence, predominantly diarrheal bowel movements, and constipation or mixed bowel habits caused by a biochemical or structural abnormality (3).

According to a systematic review, the global prevalence of IBS ranges between 9.3 and 35.5% (4). In Italy, 21.1% of medical and nursing students presented IBS based on Rome IV criteria, and it was associated with anxious symptoms and adherence to the Mediterranean diet (5). In Malta, 17.7% of medical students and young physicians experienced IBS using Rome IV criteria (6). Among French university students, IBS prevalence was 7.8%, with higher prevalence associated with depressive symptoms, stress, insomnia, cyber addiction, and eating disorders using Rome III criteria (7). In German university students, an association between psychological stress and IBS was evident according to Rome III criteria (8). In Saudi Arabia, the prevalence of IBS was 2.7% in medical students based on Rome IV criteria (9).

However, the average prevalence of IBS for Latin America was 15.4%; furthermore, according to Rome II, III, and IV criteria, the prevalence of IBS was 23.5, 11.8, and 6.98%, respectively (10). Multiple factors have been proposed that influence the increase in IBS prevalence (4), such as bacterial etiology, psychological components (stress, anxiety, and/or depression), social and academic factors, female gender (11), and disorders in eating behavior (7). In Puerto Rico, 36.3% of medical students exhibited IBS according to Rome III criteria, of which 48.1% were females with IBS, and family history of IBS and stress were positively associated with IBS, in contrast to tobacco use, which acted as a protective factor (12). In Peru, it was found that the prevalence was 9.5% for IBS, using Rome III criteria, and it was also concluded that students in their final year and those with stress symptoms were more frequently associated with this syndrome (13).

IBS represents a significant public health problem and is primarily reflected in medical students, due to their high study hour demands, academic stress overload, eating disorders, among others (4, 7). However, currently in Latin America, very little is known about the prevalence and its factors associated with IBS in this student population, as there are few published studies (11, 12). Previous studies have collected data in very small populations (14, 15). Other studies conducted on medical students in Ecuador and Peru have used outdated Rome criteria (Rome III criteria) (16, 17). Moreover, it is worth noting that the aforementioned studies have not employed rigorous biostatistical methods, the findings are descriptive, and variables such as alcohol consumption, tobacco dependence, which were associated with IBS (14–17), have not been evaluated and have been considered in the present research.

Additionally, IBS transcends its initial physiological manifestations, delving into psychosomatic intricacies that profoundly affect the enduring academic quality of life (8, 18, 19), particularly medical students. The persistent nature of IBS introduces an interplay between gastrointestinal symptoms and the psychological well-being of medical students, potentially resulting in heightened stress and anxiety over time (7, 20–22). These compounded psychosomatic effects may manifest as obstacles to sustained academic performance, disrupting concentration, productivity, and overall academic well-being specifically within the demanding context of medical education (22).

Given the above, the general objective of this study is to determine the prevalence and factors associated with IBS in Human Medicine students from a northern Peruvian university, 2021. Additionally, as a specific objective, we aim to estimate the prevalence of Irritable Bowel Syndrome (IBS) within the sampled medical student population. We hypothesize that factors such as academic year, female gender, depressive and stress symptoms, as well as eating disorders, will be associated with a higher prevalence of IBS.

## Methods

### Study design and population

An analytical cross-sectional study was conducted with the aim of determining the prevalence and factors associated with IBS in human medicine students at the Universidad San Martin de Porres (USMP)-Northern Branch during the academic period 2021–2. The study population consisted of 1,325 students from the first to the seventh academic year of the Faculty of Human Medicine at the USMP–Northern Branch during the academic semester 2021–2.

A simple random probabilistic sampling type was used, employing the statistical software EpiDat 4.2, based on a list of students enrolled in the current cycle 2021–2. For the sample size calculation, a statistical formula for finite population was used, employing a precision of 5%, a confidence level of 95%, and an expected proportion of 9.5%, similar to a study applied to human medicine students in Peru (13), resulting in a sample size of 121 students; to this, an additional 10% for losses and 10% for rejection were added, giving a sample size of 146 students. However, this research managed to capture a larger number of students than estimated, as convenience sampling was used (*n* = 409).

All students from the first to the seventh year of the Faculty of Human Medicine at the USMP-Northern Branch were included. Students who refused to participate after informed consent (*n* = 24), students who reported having a diagnosis of IBS (*n* = 4), and students who reported having a history of recently diagnosed structural gastrointestinal pathologies (less than 1 year) and/or recently performed gastrointestinal surgeries (less than 1 year) (*n* = 2) were excluded.

### Variables

IBS was the dependent variable, operationally defined as any person clinically diagnosed through a questionnaire based on the Rome IV criteria, which categorizes as IBS the presence of recurrent abdominal pain for more than 3 days per month, during the previous 3 months, associated with two or more criteria (related to defecation, changes in stool frequency, and/or changes in stool consistency).

The independent variables were age (expressed in years), sex (male or female), academic year (I, II, III, IV, V, VI, and VII year), anxiety disorders (normal, mild, moderate, severe), depression (normal, mild, moderate, severe), stress (normal, mild, moderate, severe), eating behavior disorders (present or absent), daily hours of sleep (less than 8 h/day and 8 h or more/day), self-report of frequent tobacco use (no, yes), and self-report of frequent alcohol consumption (no, yes).

### Instruments

A questionnaire was designed consisting of:

Socio-educational Data: a questionnaire was constructed based on 13 questions: academic year (from first to seventh year), age (expressed in years), sex (male or female), self-report of weight (expressed in kilograms), self-report of height (expressed in cm), self-report of frequent alcohol consumption (Yes or No), self-report of exhaustion due to academic activities (Yes or No), self-report of regular physical activity (Yes or No), quality of sleep measured by hours of sleep (<8 h/day or ≥ 8 h/day), parents’ marital status (single or married), and student’s place of residence (parents’ house, own house, or apartment rental).

Rome IV Questionnaire: designed to identify and clinically diagnose patients with the presence of Irritable Bowel Syndrome, it is composed of six questions based on the new Rome IV criteria (2). A study on the validity of this questionnaire in the adult population was conducted, considering 1,162 participants without previous gastrointestinal disorders; the questions were formulated and verified with clinical experts using a recursive process, yielding a sensitivity of 62.7% and a specificity of 97.1% for Irritable Bowel Syndrome (23). Another study conducted in Argentina compared the degree of agreement for the old and modern Rome criteria (I-IV), of which the Rome III and IV criteria stand out above the rest, having a Kappa index of 0.87, compared to Rome II (Kappa index: 0.73) and Rome I (Kappa index: 0.76) (24). The current questionnaire consists of a dichotomous question (Yes or No), a picture selection question (Bristol Scale), three multiple-choice questions: Always (100%), Almost always (66%), Sometimes (33%), and Never (0%), and the last one about frequencies (Never, less than 1 day per month, 1 day during the month, 2 to 3 days during the month, 1 day during the week, 2 to 3 days during the week, 4 to 6 days during the week, and more than 6 days during the week). To establish the diagnosis of IBS, the following must be met: Q42 (1 day a week) + Q46 (Yes) + two of the following (Q43, Q44, Q45) with a minimum response of “Sometimes.”

DASS21: questionnaire designed to measure stress, anxiety, and depression consisting of 21 items; which are subdivided into three subscales to find the aforementioned parameters (depression, anxiety, and stress). The responses are represented on a Likert scale; the options have a score of 0 (“does not describe anything that happened to me”) to three points (“Yes, this happened to me a lot, or almost always”), with the maximum score for each subscale being 21 points, respectively (25). This scale was applied in a population of adolescents and university students in Chile, yielding a Cronbach’s alpha of 0.91 (26).

SCOFF: questionnaire to determine symptoms related to eating behavior disorders (anorexia and bulimia nervosa) in the last 3 months. It consists of five items, each question has dichotomous answers (yes or no), of which affirmative answers are worth one point and negative answers zero points; thus, they will be valued on the five-point Likert scale (Between zero and five) (27). The questionnaire ranges from zero to five points, within which the cut-off points for eating behavior disorder are two or more points. In a study conducted in Colombia, in a population of young university students, it yielded a Cronbach’s alpha of 0.480 (28).

### Procedures

A virtual questionnaire was designed that included elements from the DASS-21 instruments, the SCOFF Questionnaire, and the Rome IV Criteria (obtained from the Rome Foundation) (2). This questionnaire was implemented through the REDCap platform and distributed to medical students via email and personal WhatsApp. Before participating, electronic informed consent was presented. The average time to complete the questionnaire was approximately 15 min, and its completion was promoted during weekends, avoiding coinciding with academic activities or upcoming exams. The collected data were automatically stored in a 2016 Excel database for subsequent processing and analysis.

In adherence to rigorous reporting standards, this study meticulously followed the STROBE (Strengthening the Reporting of Observational Studies in Epidemiology) checklist guidelines, ensuring transparency and completeness in presenting our observational study’s data.

### Statistical analysis

#### Statistical analysis was performed in the Stata v.17.0 program

Univariate analysis was performed to estimate absolute and relative frequencies. For numerical variables, the best measure of central tendency and dispersion was estimated, after evaluating the assumption of normal distribution. Bivariate analysis was performed, using contingency tables to evaluate the factors associated with IBS. For qualitative variables, the Chi-square test or Fisher’s exact test was used, after evaluating the assumption of expected frequencies. For numerical variables, the assumption of normal distribution was evaluated, and depending on this, the Student’s *t*-test or Mann Whitney *U* test was used, as appropriate. Finally, a multivariate analysis was conducted to determine the factors associated with IBS using simple and multiple regression models, which allowed estimating the prevalence ratio (PR) with a 95% confidence interval. Generalized Linear Models (GLM) were applied, using the Poisson distribution family, robust variance, and the academic year as a cluster. Variables that were significant in the simple model were included in the multiple model (p less than 0.05). Colinearity between the variables of interest was assessed.

## Results

### General description of the population

Out of the 403 surveyed Human Medicine students, the average age was 21 years, with a higher percentage being women (66.5%), and 22.6% were in their third year. 50.9% of the students do not exercise regularly. 77.9% reported sleeping less than 8 h a day. It was also found that 27.3% showed moderate depression, 26.5% had extremely severe anxiety, and 40.4% of the participants had eating behavior disorders. 9.7% reported having a family member diagnosed with IBS. 16.9% (95% CI: 13.37–20.86) of medical students had IBS (Table 1).

**Table 1:** Socio-educational characteristics of human medicine students.

Characteristics	N (%)
Age (years)*	21 (19–23)
Sex	
Female	272 (66.5)
Male	137 (33.5)
Academic year**	
1st year	60 (14.9)
2nd year	51 (12.7)
3rd year	91 (22.6)
4th year	60 (14.9)
5th year	40 (9.9)
6th year	39 (9.7)
7th year	62 (15.4)
Frequent alcohol consumption	
No	376 (91.9)
Yes	33 (8.1)
Frequent tobacco use	
No	392 (95.8)
Yes	17 (4.2)
Exhaustion from career activities**	
Never/almost never	15 (3.7)
Sometimes	217 (53.9)
Almost always/always	171 (42.4)
Regular exercise**	
No	198 (49.1)
Yes	205 (50.9)
Parents’ condition**	
Living together	309 (76.7)
Single	94 (23.3)
Family member diagnosed with IBS**	
No	364 (90.3)
Yes	39 (9.7)
Current housing**	
Parents’ house	351 (87.1)
Own house	23 (5.7)
Apartment rental	29 (7.2)
Level of depression**	
Normal	115 (28.3)
Mild	68 (16.8)
Moderate	111 (27.3)
Severe	50 (12.3)
Extremely severe	62 (15.3)
Level of anxiety**	
Normal	114 (28.0)
Mild	72 (17.7)
Moderate	66 (16.2)
Severe	47 (11.6)
Extremely severe	108 (26.5)
Level of stress**	
Normal	151 (37.2)
Mild	68 (16.8)
Moderate	89 (21.9)
Severe	77 (19.0)
Extremely severe	21 (5.2)
Hours of sleep**	
<8 h/day	314 (77.9)
≥8 h/day	89 (22.1)
BMI (Body Mass Index)**	
Normal	278 (69.5)
Overweight	108 (27.0)
Obesity	14 (3.5)
Eating behavior disorder**	
No	241 (59.7)
Yes	163 (40.4)
Irritable Bowel Syndrome	
No	340 (83.1)
Yes	69 (16.9)

*Median (25th percentile – 75th percentile).

**Some variables do not sum up to 409 due to missing data.

### Prevalence and characteristics of IBS

In the bivariate analysis, we found significant differences in the prevalence of IBS according to sex (10.2% males vs. 20.2% females; *p* = 0.011), age (*p* = 0.003), and academic year (*p* = 0.014). Students with severe depression levels had a higher frequency of IBS compared to those without depression (32.0% vs. 3.5%; *p* < 0.001). Students who exhibited severe anxiety levels had a higher frequency of IBS compared to those without anxiety (29.8% vs. 5.3%; *p* < 0.001). Additionally, it was found that students with eating behavior disorders had a 14.6% higher frequency of IBS compared to those without such disorders (25.8%) (Table 2).

**Table 2:** Factors associated with IBS in human medical students in bivariate analysis.

Variables	*Irritable Bowel Syndrome*		p*
	No (*n* = 340)	Yes (*n* = 69)	
	n (%)	n (%)	
Age (years)****	21 (19–24)	20 (18–22)	0.003***
Sex			0.011
Female	217 (79.8)	55 (20.2)	
Male	123 (89.8)	14 (10.2)	
Academic year			0.014
1st year	40 (66.7)	20 (33.3)	
2nd year	43 (84.3)	8 (15.7)	
3rd year	75 (82.4)	16 (17.6)	
4th year	52 (86.7)	8 (13.3)	
5th year	33 (82.5)	7 (17.5)	
6th year	34 (87.2)	5 (12.8)	
7th year	57 (91.9)	5 (8.1)	
Frequent alcohol consumption			0.834
No	313 (83.2)	63 (16.8)	
Yes	27 (81.8)	6 (18.2)	
Frequent tobacco use			0.749**
No	325 (82.9)	67 (17.1)	
Yes	15 (88.2)	2 (11.8)	
Exhaustion from career activities			0.848
Never/almost never	13 (86.7)	2 (13.3)	
Sometimes	178 (82.0)	39 (18.0)	
Almost always/always	143 (83.6)	69 (16.4)	
Regular exercise			0.302
No	168 (84.9)	30 (15.2)	
Yes	166 (81.0)	39 (19.0)	
Parents’ condition			0.364
Living together	259 (83.8)	50 (16.2)	
Single	75 (79.8)	19 (20.2)	
Family member diagnosed with IBS			0.299
No	304 (83.5)	60 (16.5)	
Yes	30 (76.9)	9 (23.1)	
Current housing			0.542
Parents’ house	289 (82.3)	62 (17.7)	
Own house	21 (91.3)	2 (8.7)	
Apartment rental	24 (82.8)	5 (17.2)	
Level of depression			<0.001
Normal	111 (96.5)	4 (3.5)	
Mild	61 (89.7)	7 (10.3)	
Moderate	84 (75.7)	27 (24.3)	
Severe	34 (68.0)	16 (32.0)	
Extremely severe	47 (75.8)	15 (24.2)	
Level of anxiety			<0.001
Normal	108 (94.7)	6 (5.3)	
Mild	67 (93.1)	5 (6.94)	
Moderate	52 (78.8)	14 (21.2)	
Severe	33 (70.2)	14 (29.8)	
Extremely severe	78 (72.2)	30 (27.8)	
Level of stress			0.092
Normal	135 (89.4)	16 (10.6)	
Mild	54 (79.4)	14 (20.6)	
Moderate	73 (82.0)	16 (18.0)	
Severe	59 (76.7)	18 (23.4)	
Extremely severe	16 (76.2)	5 (23.8)	
Hours of sleep			0.177
<8 h/day	256 (81.53)	58 (18.5)	
≥8 h/day	78 (87.6)	11 (12.4)	
BMI (Body Mass Index)			0.217
Normal	225 (80.9)	53 (19.1)	
Overweight	94 (87.0)	14 (13.0)	
Obesity	13 (92.9)	1 (7.1)	
Eating behavior disorder			<0.001
No	214 (88.8)	27 (11.2)	
Yes	121 (74.2)	42 (25.8)	

* *P*-value of categorical variables calculated with the Chi-Square test.

** *P*-value of categorical variables calculated with Fisher’s exact test.

*** *P*-value of categorical-numerical variables calculated with the Mann–Whitney U test.

**** Median – interquartile range.

In the simple regression model, we found that the factors associated with lower prevalence of IBS were age (PR: 0.88; 95% CI: 0.79–0.97) and sex (PR: 0.52; 95% CI: 0.34–0.81). Meanwhile, the factors associated with higher prevalence were having depression (PR: 6.30; 95% CI: 2.59–15.33), having anxiety (PR: 4.07; 95% CI: 1.63–10.13), and exhibiting eating behavior disorders (PR: 2.32; 95% CI: 1.70–3.18). In the multiple regression model, what was observed in the simple model was retained in terms of magnitude and direction, except for the variables of sex and anxiety. The prevalence of IBS was higher in students with depression (PR: 3.63; 95% CI: 1.26–10.49) and with eating behavior disorders (PR: 1.57; 95% CI: 1.01–2.43). Additionally, for each additional year of age, the prevalence of IBS was reduced by 9% in students (PR: 0.91; 95% CI: 0.83–0.99) (Table 3).

**Table 3:** Factors associated with IBS in human medicine students, in simple and multiple regression analysis.

Characteristics	*Irritable Bowel Syndrome*					
	Simple regression			Multiple regression		
	RP	95% CI	p*	RP	95% CI	p*
Age (years)*	0.88	0.79–0.97	0.015	0.91	0.83–0.99	0.028
Sex						
Female	Ref.			Ref.		
Male	0.52	0.34–0.81	0.004	0.85	0.61–1.18	0.335
Frequent alcohol consumption						
No	Ref.					
Yes	1.32	0.45–3.90	0.608			
Frequent tobacco use						
No	Ref.					
Yes	1.06	0.21–5.38	0.940			
Exhaustion from career activities						
Never/almost never	Ref.					
Sometimes	1.35	0.33–5.46	0.676			
Almost always/always	1.23	0.33–4.51	0.757			
Regular exercise						
No	Ref.					
Yes	1.26	0.90–1.76	0.185			
Parents’ condition						
Living together	Ref.					
Single	1.25	0.86–1.82	0.245			
Family member diagnosed with IBS						
No	Ref.					
Yes	1.40	0.72–2.71	0.317			
Current housing						
Parents’ house	Ref.					
Own house	0.49	0.17–1.39	0.179			
Apartment rental	0.98	0.44–2.16	0.952			
Depression						
No	Ref.					
Yes	6.30	2.59–15.33	<0.001	3.63	1.26–10.49	0.017
Anxiety						
No	Ref.					
Yes	4.07	1.63–10.13	0.003	1.59	0.71–3.57	0.264
Stress						
No	Ref.					
Yes	1.95	0.78–4.86	0.154			
BMI (Body Mass Index)						
Normal	Ref.					
Overweight	0.68	0.31–1.49	0.336			
Obesity	0.37	0.06–2.35	0.295			
Hours of sleep						
<8 h/day	Ref.					
≥8 h/day	0.67	0.35–1.35	0.229			
Eating behavior disorder						
No	Ref.					
Yes	2.32	1.70–3.18	<0.001	1.57	1.01–2.43	0.044

*P values obtained with Generalized Linear Models (GLM), Poisson family, log link function, robust variance, cluster by academic year.

## Discussion

### Prevalence of IBS

In our study, it was found that 16.9% of the participants had IBS, according to the new Rome IV criteria. This prevalence is within the range reported (9.3–35.5%) in a systematic review, based on 16 studies reported in 2015, in a population of human medicine students; however, the criteria used were from previous versions (Rome III, Rome II, and Rome I) (4).

Regarding European studies, our research demonstrates a prevalence lower than that found in medical and nursing students in Italy (21.1%) (5), slightly lower than reported in medical students and young physicians in Malta (17.7%) (6). However, it is higher than the prevalence described in French university students (7.8%) (7). It is important to note that our study employed the Rome III criteria for assessment (7).

Nevertheless, this is slightly higher than the average total prevalence reported for IBS in the general population in Latin America, which was estimated at 15.4% (Rome II criteria 23.5%; Rome III 11.8%; and Rome IV 6.98%) (10). The prevalence of the present study reflects a superiority compared to another study conducted in human medicine students from a university in Peru, using the Rome III criteria, in which the prevalence of IBS was 12.4% (29). It is also slightly higher than another study conducted in human medicine students from a university in Malaysia, where the Rome IV criteria were used, finding that the prevalence of IBS was 14.7% (30). However, it differs from another research carried out in human medicine students from Paraguay, using the Rome IV criteria, where the prevalence was higher (23.8%) (14). It is lower than a study conducted in human medicine students from a university in Puerto Rico; however, they used the Rome III criteria, finding a prevalence of IBS of 36.3% (12).

The high prevalence of IBS found in this research could be explained by the fact that human medicine students experience situations of high academic stress, extensive study hours that generate alterations in the circadian rhythm, leading to insomnia problems and lifestyle disorders (1). High academic stress generates stimulation in the hypothalamic–pituitary–adrenal axis, triggering the release of a series of substances, highlighting corticotropin-releasing hormone, adrenocorticotropic hormone (ACTH), and cortisol; these, in turn, affect intestinal function (inhibiting the growth of the microbiota) and stimulate the sympathetic nervous system (altering intestinal motility) (31). Lastly, it has been seen that long hours of insomnia lead to prolonged and sustained release of ACTH and cortisol, generating alterations in motility and gastrointestinal functionality, as mentioned above (32).

### Factors associated with IBS

Students with depression had a higher prevalence of IBS. This is consistent with what was described in a study conducted in human medicine students from a university in Malaysia, where it was found that students with depression were more likely to have IBS (OR: 4.7; 95% CI: 2.01–11.1) (30). Another study conducted in Human Medicine students from Arabia also found that depression increased the likelihood of IBS (OR: 3.28; 95% CI: 1.85–5.82) (33). This is consistent with a meta-analysis study that included 22 cross-sectional studies in human medicine university students, finding that students with depression had a statistical association for the development of IBS (OR: 2.15; 95% CI: 1.88–2.47) (34). Our results mirror those reported in France, where depressive symptoms were positively associated with having IBS (ORa: 1.16; 95% CI: 1.03–1.31) (7). This association could be explained by abnormal responses that occur at the level of the central nervous system (CNS) to fluctuating mood situations, which send signals to the gastrointestinal tract, leading to the appearance of intestinal contractions that subsequently cause the characteristic symptoms of IBS (30).

Having an eating disorder increased the prevalence of IBS by 57%. This is consistent with what was documented in a study conducted in France, which evaluated students from different careers (engineering, psychology, medicine, nursing, among others), and students with eating disorders were twice as likely to have IBS (OR: 2.42; 95% CI: 1.30–4.51) (7). This is reinforced by similar studies which conclude that between 41 and 52% of patients with eating disorders tend to have IBS (35–37). This association could be explained by the fact that students spend many hours studying, which leads to the development of stress that leads to the consumption of foods high in carbohydrates and fats, which contributes in the long term to the slowing of gastrointestinal transit, favoring the development of IBS with the constipation phenotype according to the Rome IV-Bristol Scale (38).

For each additional year of age, the prevalence of IBS decreased by 9%. This is consistent with what was described in a descriptive study conducted in Arabia, based on a general population (age ≥ 18 years) of 1,319 people, in which the prevalence of IBS was higher in adolescents (4.7%), but decreased with age, however, this finding was not so significant (*p* = 0.196) (39). However, it contrasts with what was reported by Vázquez R et al. who found that the prevalence of IBS was higher in senior students from Peru, compared to junior students, being statistically significant (OR: 2.77; 95% CI: 1.30–5.92; *p* < 0.01) (13).

It also contradicts the findings of Agwa et al., as their research indicated that age did not show statistical significance with IBS (*p* = 0.135) (9). Spillebout et al. similarly failed to observe an association between age and IBS in French university females (ORa: 0.98; 95% CI: 0.84–1.13) (7). This association could be explained because senior medical students have higher stress loads, however, they had more experience in managing stress loads, learned in the early years (13).

Presenting anxious symptoms increased the prevalence of IBS in the simple model, however, this association was diluted in the multiple models. This is consistent with what was described in a systematic review based on data from a university population in China, and it was found that anxiety increases the likelihood of the appearance of IBS (OR: 2.35; 95% CI: 2.03–2.72) (34). Additionally, our findings are consistent with those reported in medical and nursing students in Italy, where anxious symptoms were associated with a higher prevalence of IBS (5). This is also similar to another study in Human Medicine students from Saudi Arabia, in which it was found that anxiety increased the likelihood of presenting IBS (OR: 2.44; 95% CI: 1.30–4.55) (33). Furthermore, it is consistent with the findings reported by Spillebout et al., where French university females with anxiety had a 1.20 times higher probability of IBS (ORa: 1.20; 95% CI: 1.04–1.40) (7). However, it contrasts with what was reported by Seger et al. in Malaysia where the anxiety factor was not associated with IBS (OR:1) (30). The association found, at least in the simple model, could be explained by the same conditions that depression generates for the appearance of IBS, through which there is a bidirectional relationship between the central nervous system (CNS) and the enteric system that when faced with situations of anxiety generate abnormal responses in the motility of the gastrointestinal tract (40).

Male students had a lower prevalence of IBS in the simple model, however, this association was diluted in the multiple model. This is consistent with what was described in a study in a university population in Lebanon, in which it was found that women had a higher significant correlation with IBS compared to men (OR: 0.40; 95% CI: 0.26–0.61) (11), this factor is also reinforced with another study conducted in France, in which it was found that women were 2.4 times more likely to have IBS than men (OR: 2.4; 95% CI: 1.2–4.7) (41). Our findings align with those in France, where female university students had a 2.49 times higher probability of presenting IBS compared to males (ORa: 2.49; 95% CI: 1.14–5.45) (7). However, another study conducted in Saudi Arabia found that the male population had a higher significant correlation (OR: 3.31; 95% CI: 1.59–6.87) (42). This differs from findings in Saudi Arabian university students, where gender was not associated with IBS (*p* = 0.793) (9). This association could be explained because women experience more hormonal variations, and these hormones predispose to weaker regulation in stressful situations, in addition to hindering gastrointestinal peristalsis, as they increase sympathetic tone, and are also said to increase the perception of visceral pain; leading to a higher association with IBS compared to men (43).

### Relevance of findings in public health

The results of the present study provide preliminary scientific evidence that IBS is very common in human medicine students, thereby offering a consistent analysis for implementing preventive measures. For example, encouraging improvements in lifestyle, reinforcing participation in sports activities to reduce the constant stress experienced during university life (44, 45), or employing educational talks on nutrition such as the use of a low FODMAP diet (46) or consuming a diet richer in proteins, as it acts as a protective factor for IBS (47). All the aforementioned have had a positive effect on IBS, aiming to prevent its progression and/or severity (46). This would seek to avoid economic expenses for medical treatment and also to achieve optimal academic learning. Our study is relevant as it encourages the development of more research with a longitudinal design, in which students are followed through academic cycles, and multiple participating university campuses are included to more accurately determine the prevalence of IBS in students. Additionally, future studies could include specialized and multidisciplinary evaluation composed of gastroenterologists, psychiatrists, nutritionists, and psychologists; generating preventive measures: formulating suitable diets for each student, raising awareness of warning signs of functional dyspepsia through educational sessions to prevent the onset or progression of anxiety or depression during undergraduate studies. Thus, limiting the progression of IBS and avoiding substantial expenses in treatment.

### Practical implications of the study

The study’s revelation of a 16.9% prevalence of IBS among medical students, coupled with identified factors influencing its occurrence, holds substantial practical implications for student well-being and academic success. The lower prevalence observed among younger and male students underscores the imperative for tailored preventive and stress management strategies. Specific interventions should address the distinct challenges faced by these demographic groups, promoting a nuanced and personalized approach to enhance overall mental health and well-being. Conversely, the heightened prevalence linked to depression and eating behavior disorders necessitates the integration of mental health and nutritional support services within academic healthcare structures. These findings emphasize the imperative for medical institutions to prioritize comprehensive student wellness initiatives that address both physiological and psychological aspects. Implementing targeted interventions, including stress management programs, mental health resources, and nutritional counseling, stands poised to not only alleviate the burden of IBS but also enhance the overall health and resilience of medical students throughout their academic journey.

### Limitations and strengths

This research presents some limitations. First, potential selection bias, as it only involves findings from one university campus and the findings cannot be inferred to the entire study population. Second, due to the cross-sectional design of the study, it is not possible to attribute causality between the variables that were associated with IBS. While a longitudinal study would offer valuable insights into the long-term effects of the interventions, the current study’s cross-sectional approach provides valuable insights into the prevalence and immediate associations of Irritable Bowel Syndrome (IBS) among medical students. The findings serve as a foundation for future research endeavors, potentially including longitudinal studies, to delve deeper into the temporal aspects and causal relationships. This acknowledgment ensures transparency about the study’s scope and sets the stage for further exploration and refinement of the research questions in subsequent investigations. Third, there is a temporal bias, as it was based only on students enrolled in the study cycle, and therefore it was not possible to follow up subsequently, as the study was conducted in a specific period of time. Fourth, since the research was conducted virtually, it could lead to social desirability bias, as some students might not report real responses. Fifth, measurement bias, as variables such as sleep, physical activity, and academic stress have been measured by self-report and not with validated and reliable instruments; moreover, other variables associated with IBS have not been evaluated (family income, lifestyle characteristics, academic performance, history of traumatic events, history of intestinal infections, among others) (4, 48). Despite the limitations, we have the following strengths: this research has used updated Rome IV instruments, DASS21, which have high sensitivity and specificity, and have been applied to Human Medicine students globally (2, 16). Additionally, these findings represent a solid approach based on rigorous biostatistical-epidemiological methods that have allowed estimating consistent findings on the causative factors of IBS in human medicine university students, and have included students from all academic years. Furthermore, a large and diverse sample of the student population from a Latin American country has been collected, which adds to the existing knowledge gap in the regional context. Finally, it allows us to understand a disease that has been little reported nationally and even more so in human medicine university students.

## Conclusion

In the present study, it was found that 2 out of every 10 students developed IBS. The factors that showed a higher prevalence of association with IBS were depression and eating behavior disorders. Additionally, it was observed that as age increased, the prevalence of IBS decreased. These results underline the importance of implementing preventive measures, such as improving diet, promoting exercise, and offering educational talks, to prevent the development of IBS in this student population.

## Data availability statement

The original contributions presented in the study are included in the article/supplementary material, further inquiries can be directed to the corresponding author.

## Ethics statement

The studies involving humans were approved by the Research Ethics Committee of the Universidad de San Martín de Porres (Official Letter No. 040-2022). The studies were conducted in accordance with the local legislation and institutional requirements. The participants provided their written informed consent to participate in this study.

## Author contributions

PQ-C: Conceptualization, Data curation, Formal analysis, Investigation, Methodology, Software, Supervision, Writing – original draft, Writing – review & editing. IB-V: Conceptualization, Data curation, Formal analysis, Investigation, Methodology, Project administration, Software, Validation, Visualization, Writing – original draft, Writing – review & editing. DV-G: Data curation, Formal analysis, Investigation, Methodology, Project administration, Visualization, Writing – original draft, Writing – review & editing. VV-P: Data curation, Formal analysis, Funding acquisition, Investigation, Methodology, Project administration, Resources, Software, Supervision, Visualization, Writing – review & editing. JZ-V: Formal analysis, Methodology, Resources, Validation, Visualization, Writing – original draft. CP-V: Formal analysis, Funding acquisition, Methodology, Project administration, Resources, Supervision, Validation, Visualization, Writing – review & editing. MV-G: Data curation, Funding acquisition, Methodology, Project administration, Resources, Software, Supervision, Validation, Visualization, Writing – review & editing.
